# DDO-adjuvanted influenza A virus nucleoprotein mRNA vaccine induces robust humoral and cellular type 1 immune responses and protects mice from challenge

**DOI:** 10.1128/mbio.03589-24

**Published:** 2024-12-18

**Authors:** Victoria Gnazzo, Hanaa Saleh, Ítalo A. Castro, Adrianus C. M. Boon, Amelia K. Pinto, James D. Brien, Carolina B. López

**Affiliations:** 1 Department of Molecular Microbiology, Washington University School of Medicine, Saint Louis, Missouri, USA; 2 Center for Women’s Infectious Disease Research, Washington University School of Medicine, Saint Louis, Missouri, USA; 3 Department of Medicine, Washington University School of Medicine, Saint Louis, Missouri, USA; 4 Department of Microbiology, Immunology & Molecular Genetics, University of Kentucky, Lexington, Kentucky, USA; Monash University, Clayton, Victoria, Australia; Johns Hopkins University Bloomberg School of Public Health, Baltimore, Maryland, USA

**Keywords:** mRNA vaccine, adjuvants, type I IFN

## Abstract

A challenge in viral vaccine development is to produce vaccines that generate both neutralizing antibodies to prevent infection and cytotoxic CD8^+^ T-cells that target conserved viral proteins and can eliminate infected cells to control virus spread. mRNA technology offers an opportunity to design vaccines based on conserved CD8-targeting epitopes, but achieving robust antigen-specific CD8^+^ T-cells remains a challenge. Here, we tested the viral-derived oligonucleotide DDO268 as an adjuvant in the context of a model influenza A virus (IAV) nucleoprotein (NP) mRNA vaccine in C57BL/6 mice. DDO268 when co-packaged with mRNA in lipid nanoparticles is sensed by RIG I-like receptors and safely induces local type I interferon (IFN) production followed by dendritic cells type 1 activation and migration to the draining lymph nodes. This early response triggered by DDO268 improved the generation of IgG2c antibodies and antigen-specific Th1 CD4^+^ and CD8^+^ T-cells (IFNγ^+^TNFα^+^IL2^+^) that provided enhanced protection against lethal IAV challenge. In addition, the inclusion of DDO268 reduced the antigen dose required to achieve protection. These results highlight the potential of DDO268 as an effective mRNA vaccine adjuvant and show that an IAV NP mRNA/DDO268 vaccine is a promising approach for generating protective immunity against conserved internal IAV epitopes.

**IMPORTANCE:**

Vaccines that generate neutralizing antibodies and cytotoxic CD8^+^ T-cells targeting conserved epitopes are ideal for effective protection against viruses. mRNA vaccines combined with the right adjuvant offer a promising solution to this challenge. We show that the virus-derived oligonucleotide DDO268 enhances antibody and T-cell responses to an influenza A virus (IAV) nucleoprotein mRNA vaccine in mice. DDO268 safely induces local type I interferon production and stimulates dendritic cell activation providing enhanced protection against IAV challenge. In addition, the adjuvant activity of DDO268 allows for the use of lower antigen doses during vaccination.

## INTRODUCTION

The ideal antiviral vaccine would induce immunity against the primary target virus and its variants offering “broad” or “universal” protection. This vaccine would elicit virus-neutralizing antibodies to prevent infection and would generate type 1 cellular immunity, including antigen-specific Th1 CD4^+^ T-cells and CD8^+^ T-cells that recognize conserved viral antigens, eliminate infected cells, and prevent reinfection.

mRNA vaccines represent a milestone in vaccinology, offering safety, flexibility, cost-effective manufacturing, and rapid development (1, 2). mRNA vaccines facilitate antigen selection, allowing for easier development of viral vaccines that target conserved CD8^+^ T-cells epitopes. However, in addition to the right antigen, proper immune system direction is needed for stimulating a type 1 immune response. Type I interferons (IFNs) are key drivers of antigen-specific Th1 CD4^+^ T-cells and cytotoxic CD8^+^ T-cells during infection (3–6). In the context of vaccines, type 1 immunity can be directed by including type I IFN-inducing molecules.

mRNA vaccines consist of mRNA encoding the target protein and a lipid nanoparticle (LNP) that protects the mRNA from degradation. The LNP induces expression of chemokines and cytokines that assist with activating and recruiting immune cells to the inoculation site initiating the generation of immunity (7). The mRNA can also provide immune-activating signals, particularly when by-products of mRNA *in vitro* transcription (IVT) are present (8). These by-products can form double-stranded (ds)RNA structures that trigger type I IFN expression upon binding cellular RNA sensor proteins. To minimize excessive inflammation and adverse reactions caused by dsRNA by-products (9), mRNA vaccines are either prepared using modified nucleotides to limit their binding to RNA sensor molecules or the mRNA is purified to eliminate dsRNA products that trigger type I IFN expression (10).

Considering the above, the immunostimulatory properties of mRNA vaccines can be improved by including type I IFN-inducing molecules that can be titered for controlled type I IFN induction. We have previously identified nonstandard viral genome-derived oligonucleotides (DDOs) as effective triggers of antigen-specific type 1 immune responses during vaccination with inactivated virus or purified viral proteins. DDOs are superior to the classical adjuvants Alum and AddaVax (MF59) in initiating type 1 immunity (11, 12). DDOs are synthetic RNAs derived from a Sendai virus copy-back viral genome and activate cellular RNA viral sensors triggering type I IFN production (12). Here, we explore the use of DDOs as a type 1 immunity-inducing adjuvant for an influenza A virus (IAV) model vaccine that targets the conserved viral nucleoprotein (NP).

IAV causes approximately 3–5 million severe disease cases and 290,000–650,000 deaths annually (13). Yearly influenza epidemics and periodic pandemics result from the virus constant antigenic variation, which helps it evade host immune responses. Current IAV vaccines offer high levels of strain-specific protection but are less effective against new, antigenically distinct strains, thus requiring frequent reformulation (14). To address this problem, in addition to targeting neutralizing antigens exposed in the cell surface, IAV vaccines could be directed to the internal viral NP that is largely conserved among IAVs (15–18) and contains epitopes that are primary antigenic targets of T-cells (18).

We developed and tested a DDO268-adjuvanted mRNA vaccine encoding the IAV NP. This vaccine triggered RIG-I-like receptor signaling inducing localized type I IFN production. In addition to antibodies, the NP mRNA/DDO268 vaccine generated NP-specific CD4^+^ and CD8^+^ T-cells and protected against lethal IAV challenge. Overall, our study demonstrates that DDO268 can be harnessed as an effective inducer of type 1 immunity in the context of mRNA vaccines.

## RESULTS

### *In vivo* administration of DDO268 is safe and elicits a localized immune response without systemic effects

**FIG 1:**
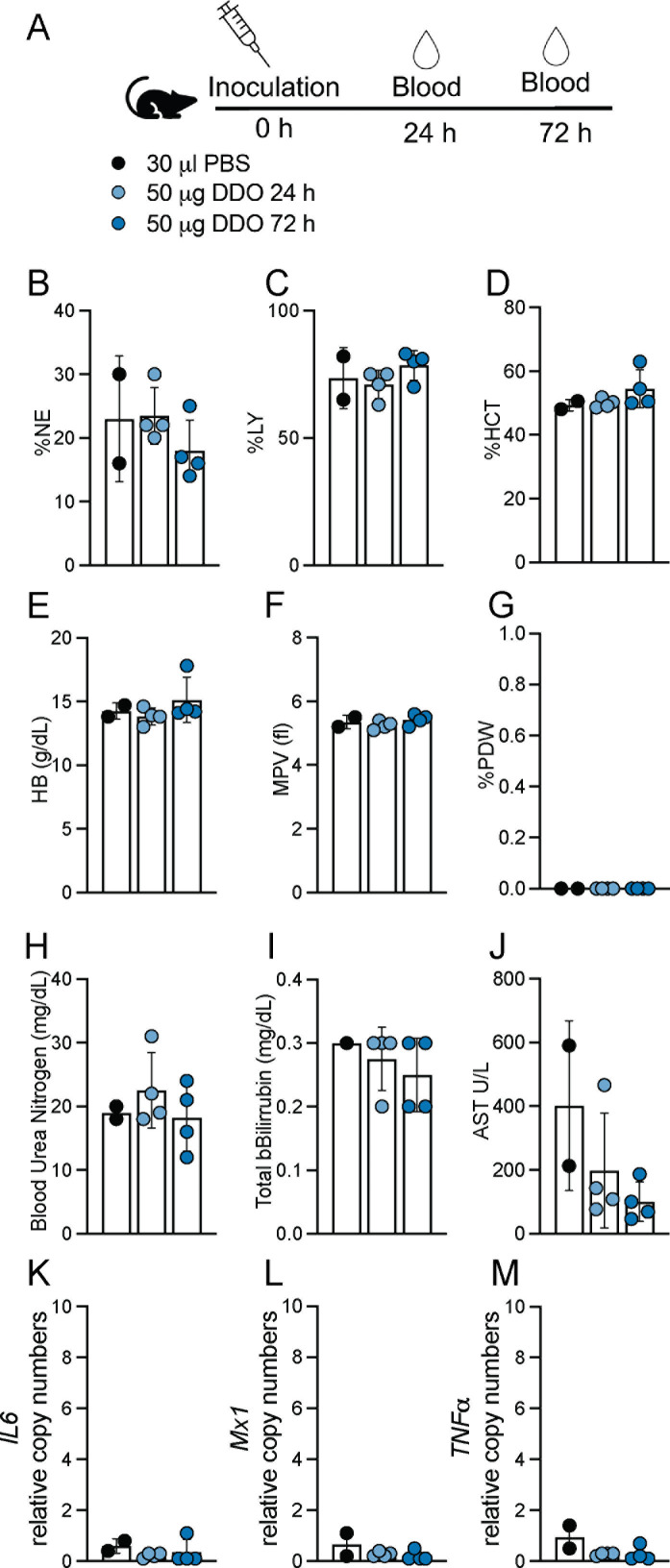
Absence of systemic toxicity after DDO268 inoculation in the footpad. (**A**) Study design. C57BL6 mice were inoculated subcutaneously with 50 µg of DDO in the rear footpad. (**B, C**) Leukogram results for percent neutrophils (%NE) and leukocytes (%LY). (**D, E**) Erythrogram results for percent hematocrit (%HCT) and hemoglobin concentration (HB g/dL). (**F, G**) Thrombocytes showing mean platelet volume (femtoliters) (MPV fl) and platelet distribution width (%PDW). (**H–J**) Chemical parameters measurements: blood urea nitrogen (BUN mg/dL); total bilirubin (mg/dL); aspartate aminotransferase (AST U/L). (**K–M**) Transcript levels of *IL6, Mx1,* and *TNFα* relative to the housekeeping genes GAPDH and β*-actin* in the liver of inoculated mice. The mean ± SD of each group is shown. *n* = 2 (mock) and 4 (treated).

We have demonstrated that DDO268 induces expression of type I IFN at the site of inoculation and not systemically (12). To further assess the safety of DDO268, we inoculated mice subcutaneously in the footpad with 50 µg of DDO268, a dose 10 times higher than that used in previous published studies (11, 12), and we evaluated complete blood counts (CBCs), serum chemistry, and systemic cytokine levels up to 72 h post-inoculation. As shown in Fig. 1 and Table S1, DDO268 did not induce significant changes in any of the measured parameters. Analysis of the leukogram, erythrogram, and thrombocyte counts (Fig. 1A through G) revealed no significant alterations in blood cell populations. Similarly, chemical parameters such as blood urea nitrogen, bilirubin, and aspartate transferase levels (Fig. 1H through J) were comparable to those in mice inoculated with PBS. Additionally, transcripts of pro-inflammatory cytokines, including *Il6* and *TNFα*, and of IFN-stimulated genes, such as *Mx1*, were not detected in the liver at any timepoint (Fig. 1K through M). These findings confirm the ability of DDO268 to safely elicit a localized immune response without detectable systemic effects.

### DDO268 promotes the generation of IgG2c antibodies and antigen-specific CD8^+^ T-cells in response to a SARS-CoV-2 mRNA vaccine

**FIG 2:**
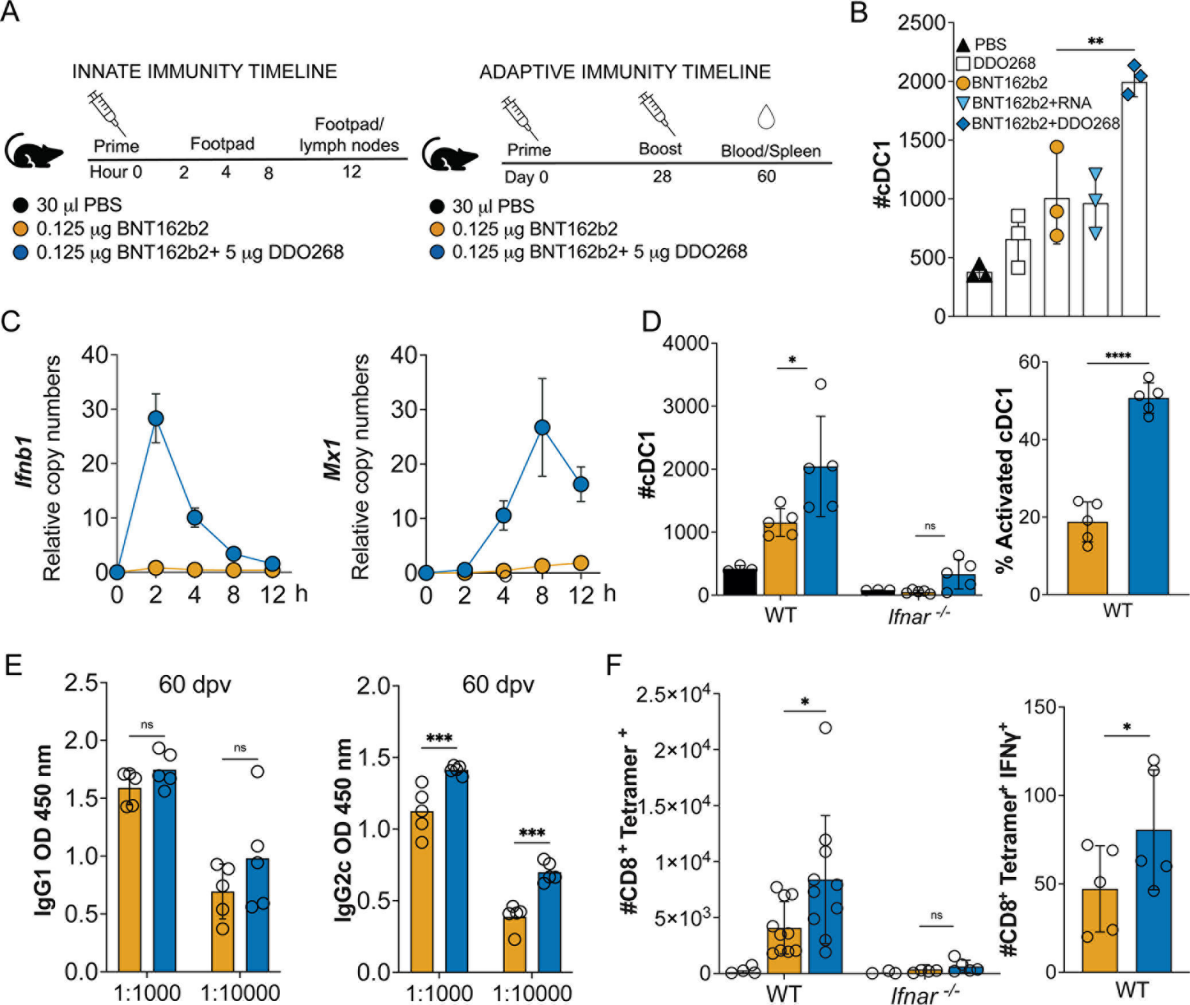
DDO268 improves type 1 immune responses to the original Pfizer SARS-CoV2 mRNA vaccine. C57BL6 WT and *Ifnar^−/−^* mice were immunized in the rear footpad with the Pfizer SARS-CoV-2 mRNA vaccine BNT162b2 (0.125 µg), or with BNT162b2 (0.125 µg) + DDO268 (5 µg). (**A**) Timeline and groups for the study design. (**B**) Number of total cDC1 in the draining lymph nodes 12 h after vaccination of C57BL6 WT mice with: PBS, DDO268 (5 µg), BNT162b2 (0.125 µg), BNT162b2 (0.125 µg) + non-immunostimulatory RNA (5 µg) or BNT162b2 (0.125 µg) + DDO268 (5 µg). cDC1 were characterized as live, CD3^−^NK1.1^−^B220^−^CD19^−^MHCII^hi^CD64^−^Ly6c^−^CD11c^hi^XCR1^+^SIRPa^−^. *N* = 3 mice per group. (**C**) *Ifnb1* and *Mx1* transcript level at the inoculation site of WT mice measured by qPCR at 2, 4, 8, and 12 h after vaccination. (**D**) Number of total and activated cDC1 in the draining lymph nodes 12 h after vaccination of WT and *Ifnar^−/^*^−^ mice. cDC1 were characterized as live, CD3^-^NK1.1^-^B220^−^CD19^−^ MHCII^hi^CD11c^hi^ CD64^−^Ly6c^−^ XCR1^+^ SIRPa^−^, activated cDC1 are live, CD3^−-^NK1.1^−^B220^−^CD19^−^ MHCII^hi^CD11c^hi^ CD64^−^Ly6c^−^ XCR1^+^ SIRPa^−^ CD86^+^. (**E**) SARS-CoV-2 Spike-specific IgG1 and IgG2c antibodies in vaccinated animals 60 days post vaccination (32 days post booster). (**F**) Number of CD8^+^ Tetramer^+^ T-cells in the spleens on day 39 after boost and specific CD8^+^ Tetramer^+^ IFNγ^+^ T-cells in the spleen. Number of cells shown was normalized to 500,000 live cells. In all experiments, the mean ± SEM of each group is shown (*n* = 3–5/group except for panel **F** WT # CD8^+^ Tetramer^+^, where data were pooled from two independent experiments. *n* = 5 mice each). * = *P* < 0.05, ** = *P* < 0.01, *** = *P* < 0.005, **** = *P* < 0.001 by unpaired *t*-test for comparisons between two groups, and one-way ANOVA or two-way ANOVA with Bonferroni’s multiple comparison test for comparisons among three or more groups.

To first evaluate the impact of DDO268 in the context of the mRNA vaccine platform, we tested its impact on the induction of type 1 immune responses during vaccination with the original severe acute respiratory syndrome coronavirus 2 (SARS-CoV-2) mRNA vaccine, BNT162b2. We vaccinated mice subcutaneously in the footpad with a simple mix of 0.125 µg BNT162b2 vaccine and 5 µg DDO268, a dose that showed no local visible inflammation, and boosted the mice 28 days later (Fig. 2A). Migration of conventional dendritic cells type 1 (cDC1) to the draining lymph node is an essential early step in the generation of type 1 immune responses upon immunization in the presence of DDO268 (12), so we first assessed cDC1 migration to the popliteal lymph node upon vaccination with either DDO268 alone, BNT162b2 alone, BNT162b2 combined with an inert RNA (a synthetic 30 nucleotide-long and non-immunostimulatory RNA), or BNT162b2 combined with DDO268. All combinations were used at equivalent molar amounts. DDO268, but not the non-immunostimulatory RNA, significantly increased the number of cDC1 in the draining lymph nodes at 12 h post vaccination (Fig. 2B; Fig. S1), a previously determined peak timepoint for cDC1 accumulation in the lymph node upon vaccination (12).

To confirm the induction of type I IFNs at the site of immunization, we measured expression of *Ifnb1* and the IFN-stimulated gene *Mx1* mRNA in the footpad after vaccine administration. DDO268 transiently and significantly enhanced the expression of these genes (Fig. 2C). In addition, in mice lacking the type I IFN receptor (*Ifnar^−/^*^−^), cDC1 migration was abolished, underscoring the role of type I IFN in the response to DDO268 (Fig. 2D). Moreover, DDO268 increased titers of circulating IgG2c antibodies (Fig. 2E) and promoted the generation of a larger population of activated SARS-CoV-2 specific CD8^+^ IFNγ^+^ T-cells in the spleen compared to BNT162b2 alone (Fig. 2F). The adaptive immune response enhancement by DDO268 depended on type I IFN signaling, similar to our previous findings with inactivated virus vaccines (12). Overall, these proof-of-principle experiments suggest that DDO268 can enhance the immune response elicited by an mRNA vaccine and bias it toward type 1 immunity in a type I IFN-dependent manner.

### Development and evaluation of an IAV NP mRNA/DDO268 vaccine

**FIG 3:**
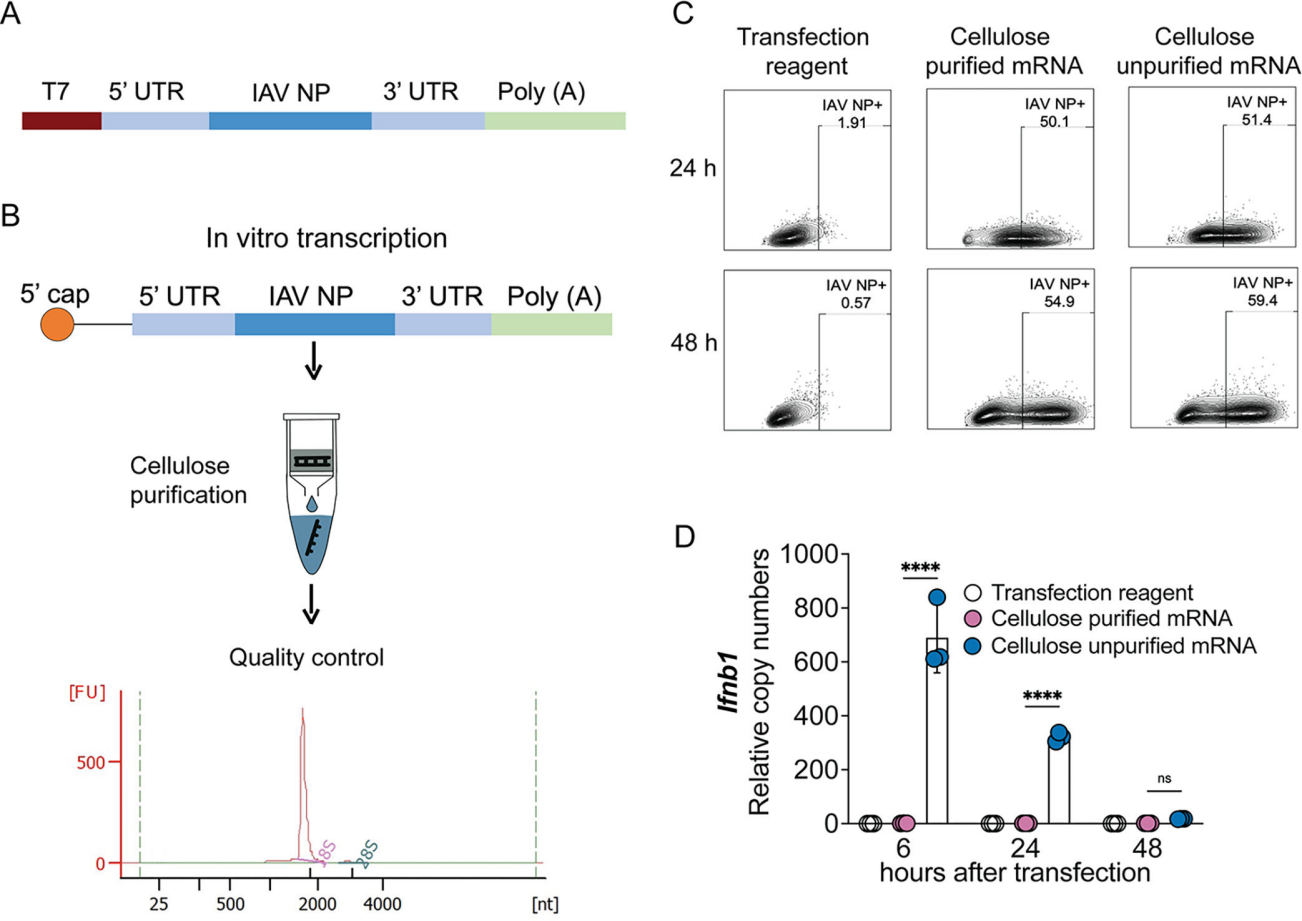
Design, characterization, and functional assessment of IAV NP mRNA. (**A**) pJB201.1 plasmid schematic. (**B**) mRNA encoding the IAV NP *in vitro* transcription and downstream process. Cellulose purification and quality control by Bioanalyzer. (**C, D**) Functional assessment of cellulose-purified and unpurified mRNA. (**C**) Comparison of IAV NP expression in A549 cells (1 × 10^6^ cells) transfected with 1 mg of cellulose purified or unpurified IVT NP mRNA at 24 and 48 h post transfection. Protein detection by flow cytometry upon intracellular staining with anti-NP antibody. (**D**) Transcript levels of *Ifnb1* relative to the housekeeping genes GAPDH and β*-actin* in A549 cells at 6, 24, and 48 h after transfection with cellulose purified and unpurified IVT NP mRNA. The mean ± SD of each group is shown (*n* = 3/group). **** = *P* < 0.001 by one-way ANOVA.

We next investigated if DDO268 promotes type 1 immunity when co-packaged with the mRNA. To this end, we developed an mRNA vaccine using IAV NP as target antigen. To eliminate unspecific immunostimulatory dsRNA, we cellulose-purified the *in vitro* transcribed unmodified IAV NP mRNA followed by stringent quality control (Fig. 3A and B). Codon-optimized influenza A/Puerto Rico/8/1934 H1N1 NP mRNA was generated from the pJB201.1 vector (Fig. 3A) and the mRNA functionality was tested in A549 cells. For the purification process, mRNA was incubated with cellulose under optimized binding conditions to ensure selective removal of double-stranded RNA while maintaining mRNA stability and functionality, followed by column purification. NP expression levels were comparable between purified and unpurified mRNAs (Fig. 3C). However, purified mRNA resulted in significantly reduced *Ifnb1* expression (Fig. 3D), confirming the effectiveness of cellulose purification in mitigating immune activation. In subsequent experiments, we used unmodified dNTPs for mRNA production followed by cellulose-purification.

**FIG 4:**
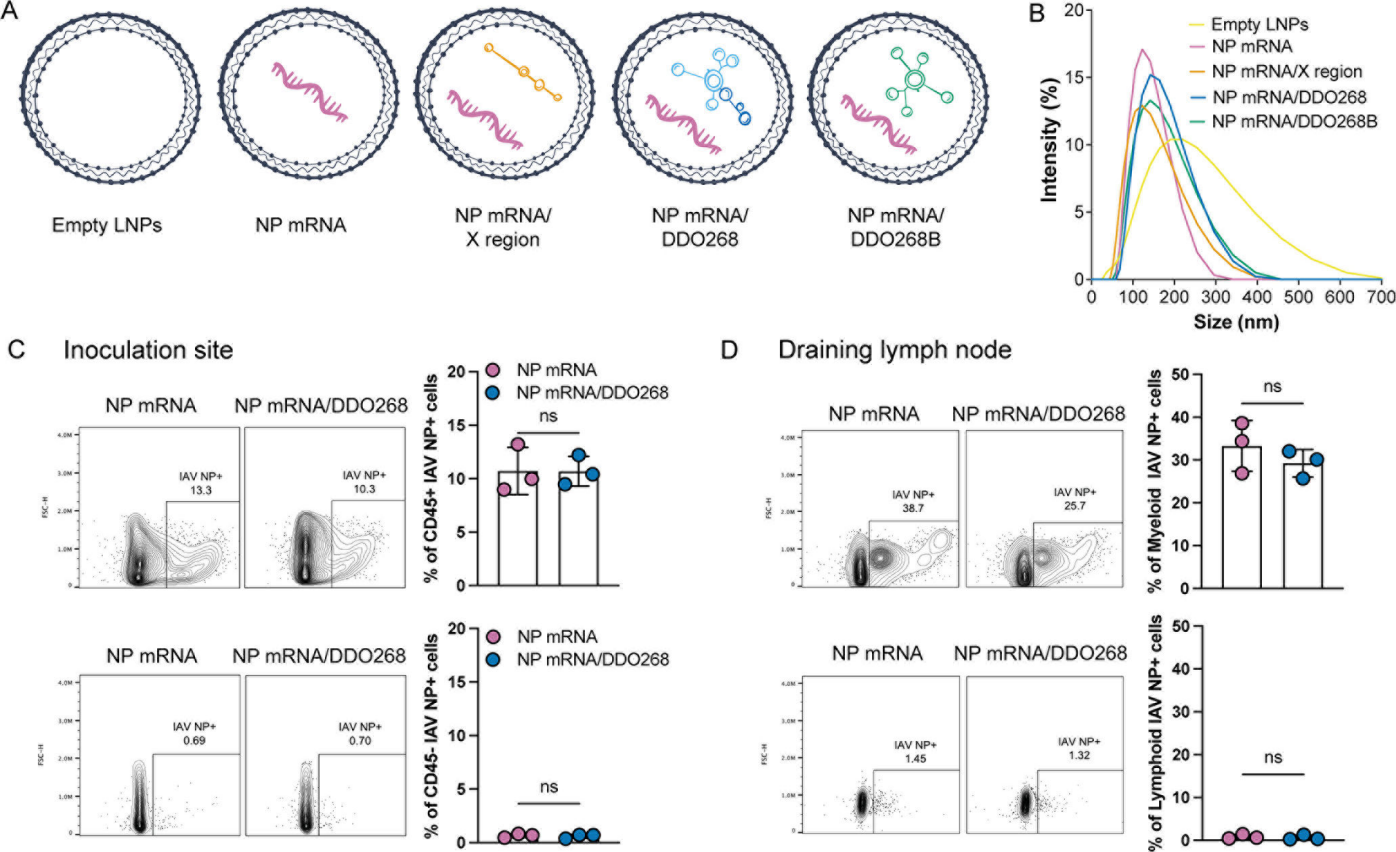
IAV NP mRNA vaccine formulation, characterization, and *in vivo* testing. (**A**) Scheme of the different vaccine formulations tested: empty LNPs; LNPs containing IAV NP mRNA, IAV NP mRNA/X region, IAV NP mRNA/DDO268, or IAV NP mRNA/DDO268B. (**B**) Size distribution of LNPs empty or carrying IAV NP mRNA, IAV NP mRNA/X region, IAV NP mRNA/DDO268, or IAV NP mRNA/DDO268B measured by dynamic light scattering. (**C, D**) Expression of IAV NP at the (**C**) inoculation site (rear footpad) or (**D**) draining lymph nodes detected by flow cytometry upon intracellular staining with anti-NP antibody 24 h after vaccination of C57BL6 mice with LNPs containing 1 µg of NP mRNA alone or 1 µg of NP mRNA/DDO268. Gating for immune cells (CD45^+^): single cells, live, CD45^+^ IAV NP^+^. Gating for nonimmune cells (CD45^−^): single cells, live, CD45^-^ IAV NP^+^. Gating for myeloid cells: single cells, live, CD3^−^ B220^−^ CD19^−^ NK1.1^−^ IAV NP^+^. Gating for lymphoid cells: single cells, live, CD3^+^ B220^+^ CD19^+^ NK1.1^+^ IAV NP^+^. The mean ± SD of each group is shown (*n* = 3/group). ns = *P* > 0.05 by unpaired *t*-test.

We formulated mRNA vaccines into LNPs using the Gen-Voy ILM reagent and co-packaged the IAV NP mRNA with a series of immunostimulatory and non-immunostimulatory RNAs. The formulations included IAV NP mRNA alone, IAV NP mRNA/DDO268, IAV NP mRNA/DDO268B (a modified DDO268 molecule, with the immunostimulatory motif encoded in reverse direction), IAV NP mRNA/X region (a non-immunostimulatory small RNA derived from the hepatitis C virus genome [19, 20]), and empty LNPs (Fig. 4A). Each formulation contained a 1:1 molar ratio of mRNA to RNA, ensuring consistent total number of RNA molecules. The RNA concentration and the encapsulation efficiency were assessed using RiboGreen RNA assay (21), and nanoparticle sizes (ranging from 120 to 150 nm) were measured by dynamic light scattering (DLS) (Fig. 4B).

To assess protein expression and identify the cell types expressing the viral protein *in vivo*, we inoculated mice subcutaneously and evaluated NP expression at the inoculation site and in the draining lymph nodes at 24 h post-vaccination. NP was expressed in CD45^+^ cells in the footpad (Fig. 4C) and in myeloid cells within draining lymph nodes (Fig. 4D). These results agree with previous reports of mRNA vaccines being primarily taken up by immune cells at the site of immunization (22–24).

### DDO is sensed by intracellular RIG-I-like receptors when packaged within LNPs

**FIG 5:**
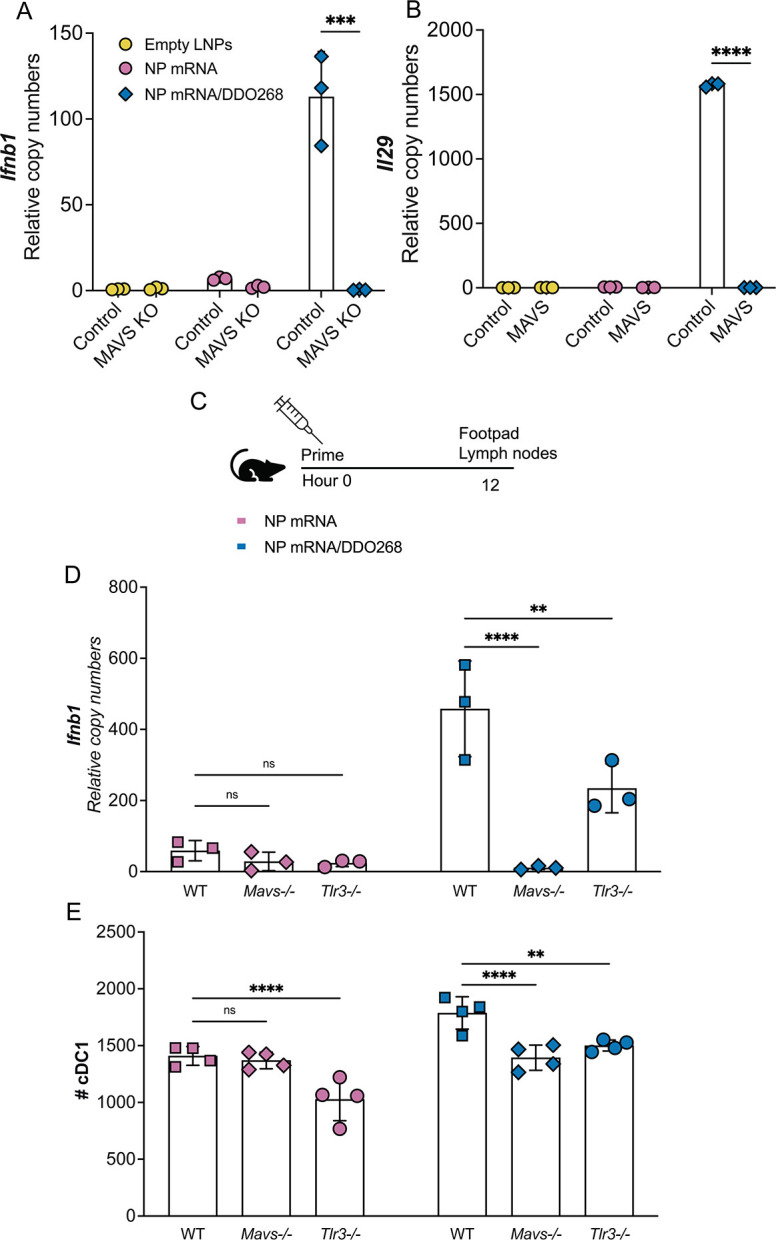
DDO268 within LNPs is detected by RIG-I. (**A, B**) A549 control and MAVS KO cells were transfected with LNPs containing 0.5 µg of NP mRNA alone or NP mRNA/DDO268. Transcript levels of (**A**) *Ifnb1* and (**B**) *Il29* relative to the housekeeping genes GAPDH and β*-actin* at 24 h after transfection. (**C**) *In vivo* study design. C57BL6 WT, *Mavs^−/^*^−^ and *Tlr3^−/^*^−^ mice were immunized in the rear footpad with LNPs containing 0.5 µg of NP mRNA alone or 0.5 µg of NP mRNA/DDO268. (**D**) Transcript levels of *Ifnb1* relative to the housekeeping genes GAPDH and β*-actin* in the footpad of C57BL6 mice 12 h after vaccination. (**E**) Number of cDC1 in the draining lymph nodes 12 h after vaccination (mean ± SEM of each group is shown). (*n* = 3–4/group). ** = *P* < 0.01, *** = *P* < 0.005, **** = *P* < 0.001 by two-way ANOVA with Bonferroni’s multiple comparison test.

We have reported that DDO delivered subcutaneously in its naked form triggers signaling by the cell-type-restricted endosomal RNA sensor TLR3 (12). However, when transfected into cells, DDO is sensed by cytoplasmic RIG-I-like receptors (20). We hypothesized that DDO delivered within LNPs would be detected by the ubiquitously expressed intracellular RNA sensors, providing the advantage of adjuvant functionality in a much larger population of target cells. To test this hypothesis, we compared LNPs containing IAV NP mRNA alone or LNPs containing IAV NP mRNA/DDO268 for their ability to trigger type I IFN expression in cells lacking RIG-I signaling (MAVS KO) or control cells. As expected, DDO induced high levels of MAVS-dependent transcription of *Ifnb1* and *IL29* (Fig. 5A and B).

To determine if encapsulated DDO triggers RIG-I -like receptors *in vivo*, we inoculated control, *Mavs^−^*^/−^, or *Tlr3^−^*^/−^ mice and tested type I IFN expression at the injection site, as well as cDC1 cell accumulation in the draining lymph node 12 h later (Fig. 5C). In WT mice, the DDO-adjuvanted vaccine induced high levels of *Ifnb1* while significantly lower levels of *Ifnb1* were detected in *Mavs^−^*^/−^ mice (Fig. 5D). *Tlr3^−^*^/−^ mice showed a moderate decrease in *Ifnb1* expression in both formulations, suggesting that TLR3 is not sufficient nor necessary for DDO adjuvancy in this context. These findings provide two critical insights: (i) cells detect and respond to DDO268 when encapsulated in LNPs; and (ii) DDO268 induces type I IFN expression necessary to initiate a type 1 immune response in a RIG-I-dependent manner.

### DDO268 confers type I IFN-inducing ability to the IAV NP mRNA vaccine

**FIG 6:**
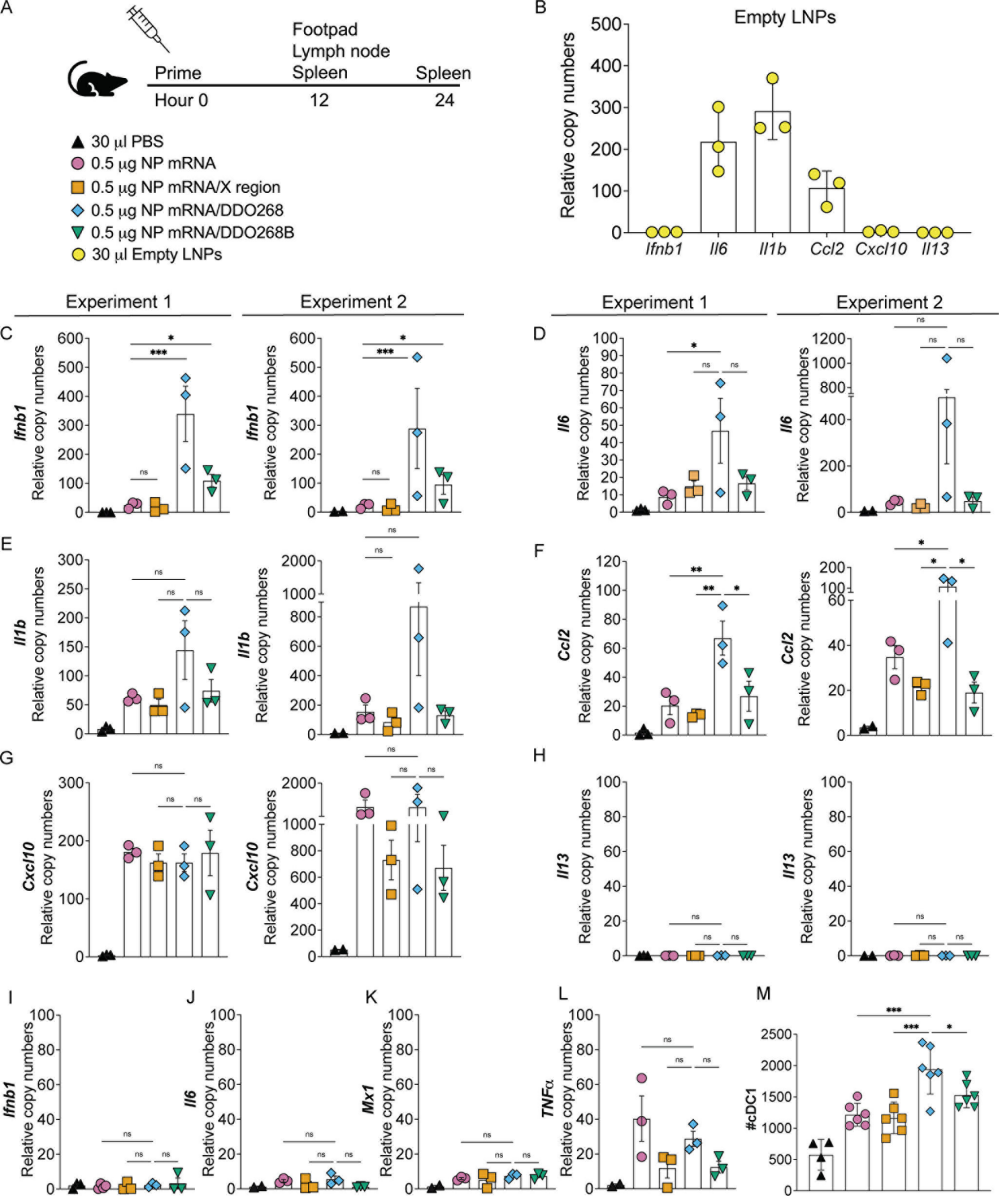
DDO268 promotes strong type 1 innate immune responses to the IAV NP mRNA vaccine. (**A**) Timeline and groups for the study design. (**B**) Transcript levels of *Ifnb1*, *Il6, Il1b*, *Ccl2*, *Cxcl10,* and *Il13* relative to the housekeeping genes GAPDH and β*-actin* in the footpad of C57BL6 mice 12 h after vaccination with empty LNPs. Transcript levels of (**C**) *Ifnb1*, (**D**) *Il6,* (**E**) *Il1b*, (**F**) *Ccl2*, (**G**) *Cxcl10,* and (**H**) *Il13* relative to the housekeeping genes GAPDH and β*-actin* in the footpad of C57BL6 mice 12 h after vaccination with PBS; LNPs containing 0.5 µg of NP mRNA, NP mRNA/X region, NP mRNA/DDO268, or NP mRNA/DDO268B. (**I**) Transcript levels of *Ifnb1* in the spleen of C57BL6 mice 12 h after vaccination with PBS; LNPs containing 0.5 µg of NP mRNA or 0.5 µg NP mRNA/X region; 0.5 µg NP mRNA/DDO268; 0.5 µg NP mRNA/DDO268B. Transcript levels of (**J**) *Il6,* (**K**) *Mx1,* and (**L**) *TNF*α in the spleen of C57BL6 mice 24 h after vaccination with PBS; LNPs containing 0.5 µg NP mRNA, NP mRNA/X region, NP mRNA/DDO268, or NP mRNA/DDO268B. (**M**) Number of cDC1 in the draining lymph nodes 12 h after vaccination with PBS; empty LNPs; LNPs containing 0.5 µg NP mRNA, NP mRNA/X region, NP mRNA/DDO268, or NP mRNA/DDO268B. cDC1 were characterized as live, CD3^−^NK1.1^−^B220^−^CD19^−^ MHCII^hi^CD11c^hi^ CD64^−^Ly6C^−^ XCR1^+^SIRPa^−^. Mean ± SEM of each group is shown (*n* = 3/group). *P < 0.05, **P < 0.01, ***P < 0.005, by one-way ANOVA with Bonferroni’s multiple comparison test. (**C–H**) Two independent experiments are shown (*n* = 3 mice each). (**M**) Data represent two independent experiments where data were pooled with *n* = 3 mice, *n* = 2/3 for the PBS group.

To confirm that our vaccine induced local expression of type I IFN, cytokines, and chemokines, we analyzed footpads and popliteal lymph nodes 12 h after subcutaneous inoculation, as well as spleens at 12 and 24 h after subcutaneous inoculation (Fig. 6A). As expected, empty LNPs induced *Il1b*, *Il6,* and *Ccl2* expression (7) but showed minimal *Ifnb1* or *Cxcl10* expression at the inoculation site (Fig. 6B). LNPs containing IAV NP mRNA or IAV NP mRNA/X region induced low *Ifnb1* levels, while the LNPs containing IAV NP mRNA/DDO268B showed higher levels than controls but lower than the DDO268 formulation (Fig. 6C). All vaccine formulations induced *Il6*, *Il1b, Ccl2*, and *Cxcl10* transcription (Fig. 6D through G). Transcripts of *Il13,* a type 2 immunity-associated cytokine were undetected at the inoculation site for all formulations (Fig. 6H). Importantly, DDO268-containing formulations did not induce significant *Ifnb1* in the spleen at 12 h after vaccination (Fig. 6I) or *Il6*, *Mx1*, and *Tnfα* expression in the spleen at 24 h after vaccination (Fig. 6J through L), confirming that DDO268 adjuvancy induces a localized immune response (12). Additionally, cDC1 recruitment to the draining lymph nodes increased significantly with DDO268 (Fig. 6M). These findings confirm that the DDO268 enhances type I IFN expression without triggering systemic inflammation.

### IAV NP mRNA/DDO268 vaccine elicits antigen-specific type 1 humoral and cellular immune responses

**FIG 7:**
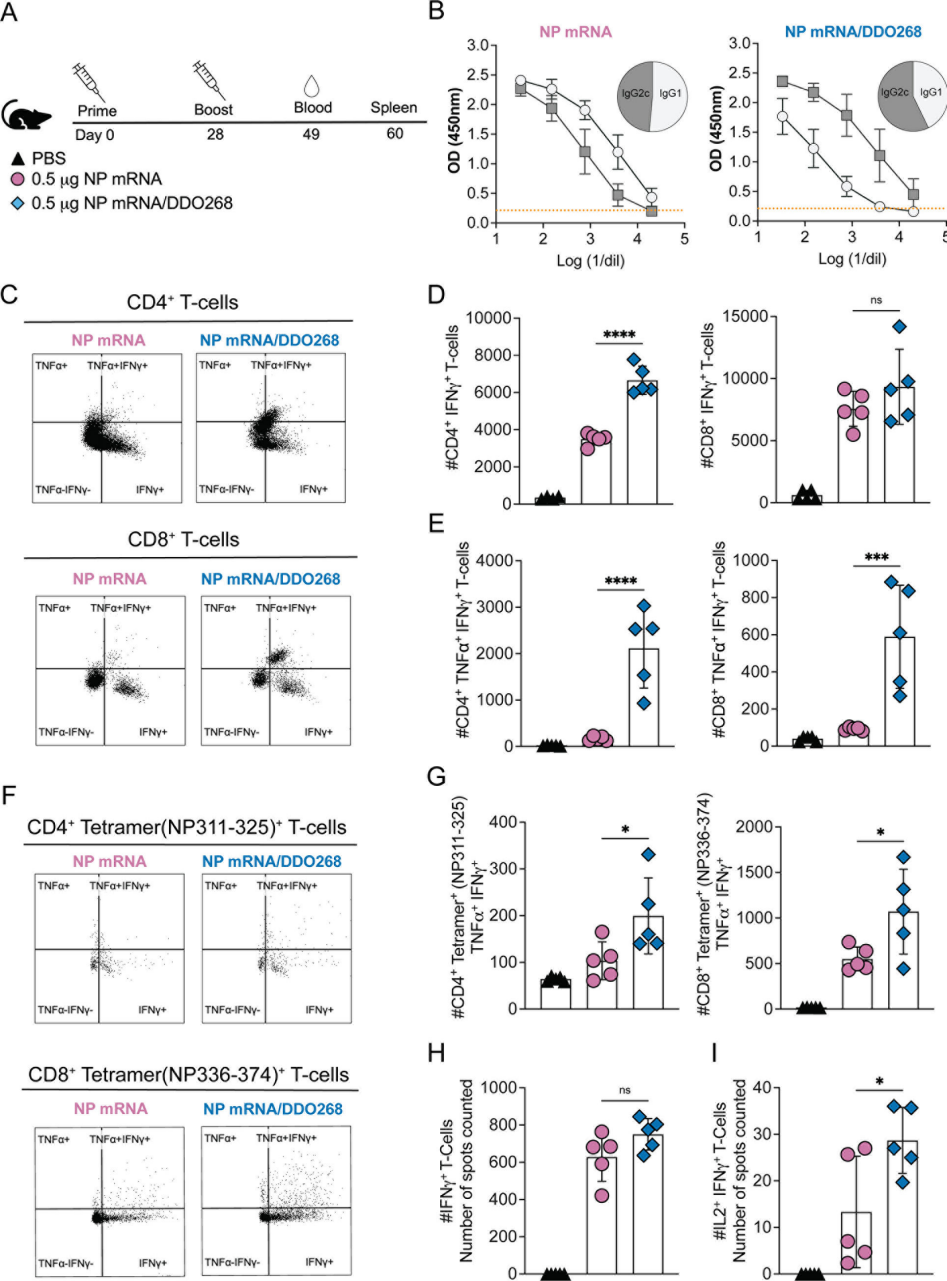
DDO268 promotes strong type 1 adaptive humoral and cellular immune responses to the IAV NP mRNA vaccine. (**A**) Timeline and groups for the study design. C57BL6 mice were immunized twice 28 days apart with LNPs containing 0.5 µg NP mRNA or NP mRNA/DDO268. (**B**) Blood was collected 3 weeks after booster immunization and specific NP antibodies IgG1 and IgG2c subtypes were evaluated by ELISA. Sera were serially diluted, and the orange line corresponds to the cutoff (normal mouse serum OD + 2 DS). The pie graphs represent the ratio of IgG1 and IgG2c in mouse serum at a dilution of 1:32. (**C–I**) Antigen-experienced cells in the spleen were examined on day 32 after the booster immunization. (**C**) Representative flow cytometry plots for CD4^+^ and CD8^+^ TNFα^+^ IFNγ^+^ from the spleens of vaccinated mice. CD4^+^ TNFα^+^ IFNγ^+^ were identified by gating on live, singlets, CD3^+^ CD4^+^ CD8^−^ CD11a^+^ cells. CD8^+^ TNFα^+^ IFNγ^+^ were identified by gating on live, singlets, CD3^+^, CD8^+^ CD4^−^ CD11a^+^ cells. (**D**) Number of CD4^+^ IFNγ^+^ and CD8^+^ IFNγ^+^ T-cells in the spleens of individual mice in each vaccination group after Ionomycin/PMA restimulation. (**E**) Number of CD4^+^ TNFα^+^ IFNγ^+^ and CD8^+^ TNFα^+^ IFNγ^+^ T-cells in the spleens of individual mice in each vaccination group after Ionomycin/PMA restimulation. (**F**) Representative flow cytometry plots for CD8^+^ Tetramer (NP336-374)^+^ and CD4^+^ Tetramer (NP311-325)^+^ TNFα^+^ IFNγ^+^ T-cells. Specific CD4^+^ TNFα^+^ IFNγ^+^ were identified by gating on live, singlets, CD3^+^, CD4^+^ CD8^−^, Tetramer (NP311-325)^+^. CD8^+^ TNFα^+^ IFNγ^+^ were identified by gating on live, singlets, CD3^+^, CD8^+^ CD4^−^, Tetramer (NP336-374)^+^. (**G**) Number of Tetramer-specific CD4^+^ Tetramer (NP311-325)^+^ TNFα^+^ IFNγ^+^ and CD8^+^ Tetramer (NP336-374)^+^ TNFα^+^ IFNγ^+^ in the spleens of individual mice in each vaccination group after specific IAV NP restimulation. Number of cells shown was normalized to 500,000 live cells. (**H**) Number of spots counted as IFNγ^+^ after specific IAV NP restimulation (from 200,000 cells). (**I**) Number of spots counted as double-positives for IL2 and IFNγ after specific IAV NP restimulation (from 200,000 cells). Data correspond to individual mice with mean ± SEM (*n* = 5/group). *P < 0.05, ***P < 0.005, ****P < 0.001 by one-way ANOVA with Bonferroni’s multiple comparison test.

We next evaluated the adaptive immune responses elicited by the IAV NP mRNA/DDO268 vaccine after primary vaccination and a booster immunization administered 4 weeks later (Fig. 7A). We also tested DDO268B, which triggers less robust responses (Fig. 6; Fig. S2). Three weeks post-booster, anti-NP IgG1 and IgG2c antibodies were measured from sera to determine Th2- and Th1-biased responses, respectively. The IAV NP mRNA vaccine induced higher IgG1 than IgG2c levels, while the IAV NP mRNA/DDO268 or DDO268B vaccine induced higher IgG2c levels, indicating a Th1-biased humoral response (Fig. 7B; Fig. S2B). These results align with our findings of enhanced IgG2c antibodies to an inactivated vaccine when DDO268 was added as an adjuvant (11, 12).

To assess T-cell responses, mice were euthanized 32 days post-booster immunization and their splenocytes were *in vitro* restimulated with ionomycin/phorbol myristate acetate (PMA). Both mRNA vaccines induced CD4^+^IFNγ^+^ and CD8^+^IFNγ^+^ T-cells in the spleen (Fig. 7C and D). However, the DDO268-adjuvanted NP mRNA vaccine significantly increased numbers of double-positive (TNFα^+^IFNγ^+^) CD4^+^ and CD8^+^T-cells (Fig. 7C and E), suggesting that DDO268 facilitates T-cell activation. As IAV NP contains T-cells epitopes (25, 26), we also evaluated the induction of IAV-specific CD4^+^ T-cells and CD8^+^ T-cells. Both IAV NP mRNA and IAV NP mRNA/DDO268 or DDO268B formulations induced NP-specific CD8^+^ and CD4^+^ T-cells in the spleen. After *in vitro* IAV NP restimulation, the DDO268-adjuvanted mRNA vaccine induced significantly higher populations of IAV-specific CD8^+^ Tetramer (NP336-374)^+^ and CD4^+^ Tetramer (NP311-325)^+^ double-positive for TNFα^+^IFNγ^+^ (Fig. 7F and G). Additionally, a two-color (IFNγ and IL2) ELISpot assay showed higher double-positive spots for IFNγ and IL2 (Fig. 7I; Fig. S2F) in the DDO268 (or DDO268B)-containing formulation after *in vitro* restimulation. Overall, DDO268-adjuvanted formulations induced CD8^+^ and CD4^+^ T-cells characterized by both IFNγ and TNFα or IL2 production.

### IAV NP mRNA/DDO268 vaccine protects mice against IAV challenge

**FIG 8:**
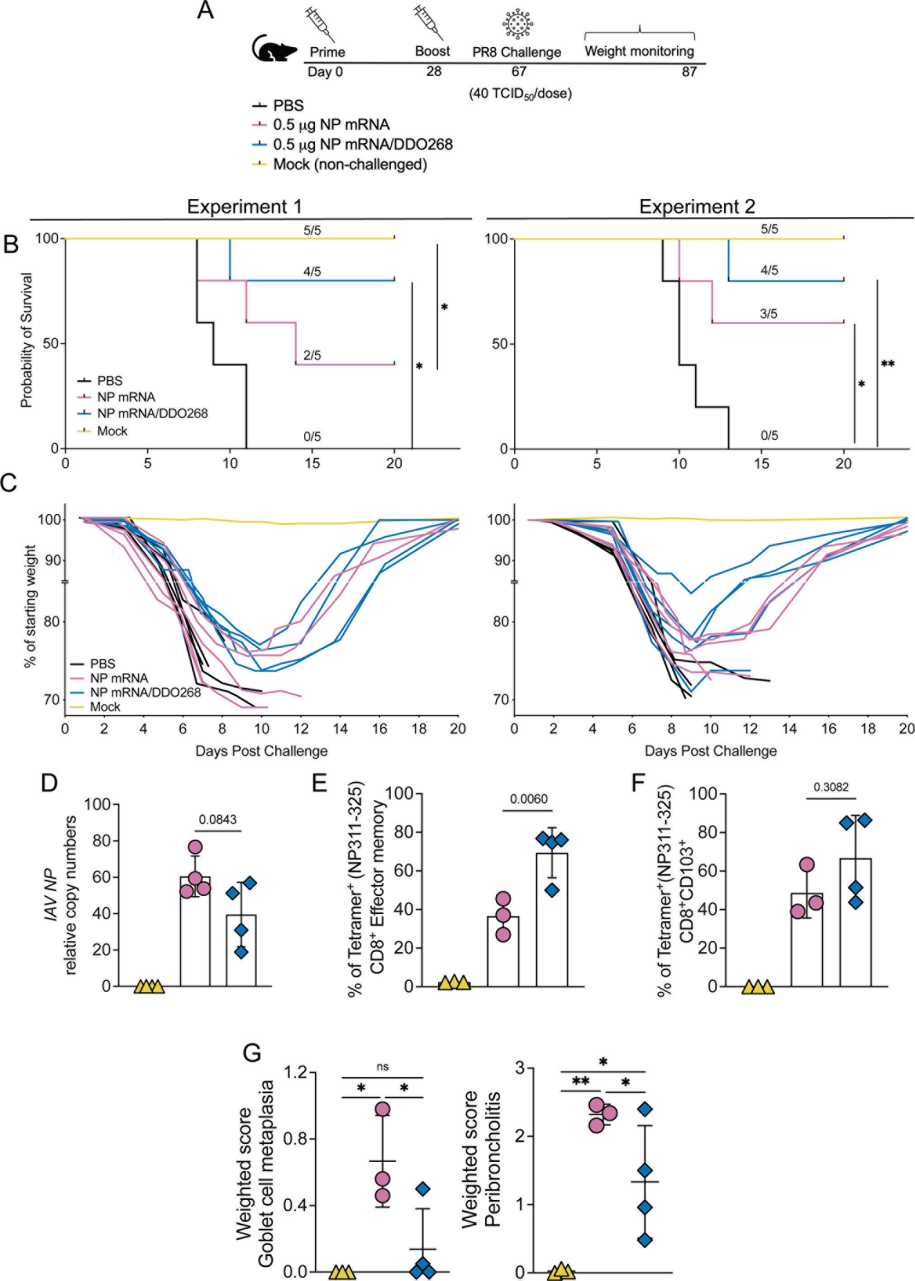
DDO268 enhances protective immunity against Influenza A/PR8 following IAV NP mRNA vaccination. (**A**) Timeline and groups for the study design. Vaccinated C57BL6 mice were challenged with 40 TCID_50_/dose of mouse passaged PR8 (H1N1) intranasally on day 39 after boost immunization. (**B**) Probability of survival in two independent experiments, *n* = 5 mice each. (**C**) Weight relative to initial body weight over time in two independent experiments, *n* = 5 mice each. (**D–F**) Data correspond to experiment 2, *n* = 3–5 (surviving animals). (**D**) Transcript levels of *IAV NP* relative to the housekeeping genes GAPDH and β*-actin* in the lungs of vaccinated mice at 7 days after challenge. (**E**) Percentage of effectors CD8^+^ in the lungs that are Tetramer (NP336-374)^+^ at day 24 post challenge determined by staining and flow cytometry analysis. Effector Tetramer^+^ cells were identified after gating on single cells, live, CD3^+^, CD8^+^, CD44^+^, CD62L^−^, CCR7^−^, Tetramer (NP336-374)^+^. (**F**) Percentage of CD8^+^ CD103^+^ T-cells in the lungs that are Tetramer (NP336-374)^+^ at day 24 post challenge determined by staining and flow cytometry analysis (identified after gating on single cells, live, CD3^+^, CD8^+^, CD44^+^, CD103^+^, Tetramer (NP336-374)^+^. (**G**) Lung sections from vaccinated and challenged survivors mice were blindly scored for histopathological changes. Weighted score for goblet cell metaplasia and peribronchiolitis were determined for every individual lung sample. * = *P* < 0.05, ** = *P* < 0.01, as determined by log-rank Mantel-Cox test for survival and by one-way ANOVA with Bonferroni’s multiple comparison test.

To evaluate the protective efficacy of our mRNA vaccines against challenge with a lethal dose of IAV PR8 (H1N1), mice were challenged intranasally with 40 TCID_50_ of IAV PR8 39 days after booster immunization and monitored for weight loss for 20 days (Fig. 8A). In two independent experiments, mice inoculated with the IAV NP mRNA/DDO268 vaccine had a higher survival probability after a lethal dose of virus than mice vaccinated with LNPs containing IAV NP mRNA (Fig. 8B). Although all mice experienced bodyweight loss (Fig. 8C), the DDO268-containing formulation induced more robust specific type 1 immune response, effectively protecting against IAV infection. Viral RNA transcripts were assessed 7 days post challenge (Fig. 8D) with *IAV NP* transcripts detected in both groups but reduced in mice vaccinated with IAV NP mRNA/DDO268. At the conclusion of the experiments, mice were sacrificed, and the presence of IAV NP-specific memory effectors CD8^+^ and CD103^+^ CD8^+^ T-cells in the lungs was evaluated. As shown in Fig. 8E and F, both vaccines generated a population of effector memory CD8^+^ Tetramer^+^ (Fig. 8E) and CD8^+^ Tetramer^+^ CD103^+^ (Fig. 8F). However, the percentage of these cells was higher in mice vaccinated with IAV NP mRNA/DDO268, indicating stronger protective immune response elicited by the DDO-adjuvanted vaccine. Moreover, the DDO-containing formulation reduced lung pathology in challenged mice compared with vaccine lacking DDO268 (Fig. 8G).

## DISCUSSION

A significant challenge in developing vaccines that protect against intracellular pathogens is the lack of appropriate and safe adjuvants to drive robust type 1 cellular immunity. The ideal type 1 adjuvant would safely stimulate a broad range of cell types. Our lab identified DDO268, which induces type I IFNs and proinflammatory cytokines by stimulating the cellular sensors RIG-I (20) and TLR3 (12), as a candidate type 1 immunity-inducing adjuvant. DDO268 is a synthetic and replication-incompetent RNA derived from the 546-nucleotide-long Sendai virus nonstandard viral genome, which is a primary immunostimulatory molecule during infections (27, 28). Results presented here together with earlier research (12) provide evidence that *in vivo* administration of DDO is safe and elicits a localized immune response without systemic effects (Fig. 1).

We have reported that DDO mixed with a purified protein antigen or an inactivated virus vaccine triggers TLR3 signaling (12). TLR3, which is expressed in antigen-presenting cells, recognizes RNA and signals for type I IFN production. However, supporting previous data (20, 29), here we show that when DDO is delivered intracellularly, it activates RIG-I-like receptors. One significant advantage of RIG-I stimulation is its close to universal expression in animals and in most nucleated cells, increasing the chances that the adjuvant activity of DDO will be conserved across model animals and humans.

Most mRNA vaccine efforts focus on antibody-mediated protection due to the crucial role of antibodies in neutralizing extracellular pathogens. However, antibodies are generally insufficient against intracellular pathogens. Current human IAV vaccines target the hemagglutinin protein (HA), which is not conserved across all strains. To develop a universal influenza vaccine, strategies have been explored to improve cross-reactive immunity against HA. One approach targets the more conserved stalk region of HA and has progressed to clinical trials. While these strategies induce antibodies that cross-react with multiple subtypes and protect against mortality, significant morbidity still occurs after infection (reviewed in references 30, 31). T-cells recognize epitopes in the NP of influenza viruses, which remains largely conserved despite antigenic shift (32, 33). Our data show that NP-targeted immunization can protect animals from severe disease and death, suggesting that NP-targeted vaccines can complement vaccines aimed at generating neutralizing antibodies for additional protection.

In this study, we used purified mRNA, ensuring that DDO was the primary source of type I IFN in the formulated vaccine. We used unmodified dNTPs, as methyl-pseudouridine, commonly used to enhance mRNA stability (34), can lead to translational errors or “slippage,” causing aberrant proteins and immune recognition (35). Our data show that mRNA purification is a valid alternative to modified NTPs when it comes to controlling excessive inflammation.

LNPs act as adjuvants for mRNA vaccines, enhancing the immune response by inducing the production of *Il6, Il1b,* and *Ccl2* (7), which recruit immune cells to the inoculation site. However, production of type I IFN is primarily driven by the mRNA (7). Our results corroborate these findings, as we observed significant induction of *Il6, Il1b,* and *Ccl2* but no induction of *Ifnb1, Cxcl10,* or *Il13* when empty LNPs were used. Additionally, we observed minimal local induction of *Ifnb1* during immunization with IAV NP mRNA and IAV NP mRNA/X region (Fig. 6C) compared with the IAV NP mRNA/DDO268 formulation, demonstrating that most IFN induction is attributed to DDO268. These results highlight the advantages of our DDO-containing formulation, which includes a controlled, local, and transient type I IFN induction.

Our data agree with our previous findings (12) showing that type 1 immunity induced by DDO268 in mice requires localized type I IFN expression and the migration of conventional dendritic cells type 1 (cDC1) to the draining lymph nodes. cDC1 cross-present antigens play a critical role in generating CD8^+^ T-cell responses to subunit and killed vaccines (36). Here, we demonstrate that the IAV NP mRNA/DDO268 vaccine enhances cDC1 activation and migration, subsequently priming CD8^+^ T-cells. DDO268-adjuvanted formulation also increases IgG2c levels in vaccinated mice (Fig. 7B) aligning with our previous reports (11, 12) where DDO268 enhanced IgG2c production in protein and purified virus vaccines. The higher IgG2c levels compared with the unadjuvanted vaccine suggest a skewing of the immune response toward a type 1 profile. Consistently, the DDO268-adjuvanted vaccine induced a higher population of fully activated NP-specific T-cells, as evidenced by increased IFNγ^+^TNFα^+^ or IFNγ^+^IL2^+^ production (Fig. 7). This enhanced activation profile highlights the effectiveness of DDO268 in boosting the cellular immune response, crucial for long-term protection against viral infections.

The enhanced immune response generated by the DDO268-adjuvanted mRNA vaccine is evidenced by increased survival of mice following a lethal IAV PR8 infection (Fig. 8). Protection was observed even at a lower vaccine dose (0.5 µg) compared with the standard 5 µg dose for mRNA vaccines. Our findings suggest that the DDO268 adjuvant not only enhances the immune response toward type 1 immunity but also allows to reduce the antigen dose required for protection, contributing to the vaccine effectiveness against IAV.

Overall, this study highlights the ability of DDO268 to promote robust T-cell and humoral immunity to conserved viral antigens, emphasizing its potential as a valuable component in mRNA vaccine formulations. Future research will explore the application of DDO268 in other vaccine platforms for infectious diseases and cancer.

## MATERIALS AND METHODS

### Mice

C57BL/6 mice, *Mavs^−^*^/^*^−^, Tlr3^−^*^/−^ (The Jackson Laboratory), and *Ifnar1^−^*^/−^ (provided by Dr. Thomas Moran) (37) were bred in-house. All mice were sex and age matched. Both male and female mice were included in the experiments.

### Viruses

IAV/Puerto Rico/8/1934 H1N1 was used as challenge strain. IAV was grown in 10-day-old embryonated chicken eggs as described in dx.doi.org/10.17504/protocols.io.5jyl8dm79g2w/v1.

### mRNA production

The mRNA construct encodes the NP of IAV/Puerto Rico/8/1934 H1N1 (GenBank: ACV49549.1). The codon-optimized sequence (Twist Bioscience) was cloned into the mRNA production plasmid (pJB201.1) that contains a T7 type II promoter, the beta globin 5′ UTR, a Kozak sequence with an AG mutation allowing for 5′ capping, and the alpha globin 3′ UTR followed by a 142 base pair poly(A) tail. mRNA was produced as described in doi.org/10.17504/protocols.io.e6nvw11d7lmk/v1. mRNA size and integrity were assessed in a Agilent Bioanalyzer 2100. dsRNA contaminants were removed following the Baiersdorfer protocol (9) as described in dx.doi.org/10.17504/protocols.io.n2bvjnn3wgk5/v1.

### mRNA construct testing

IVT mRNA was tested in HEK-293t and A549 cells using jetMESSENGER (Polyplus) as transfection reagent. IAV NP production from the IVT mRNA was measured 24 and 48 h after transfection by intracellular staining using NP IAV antibody NBP3-12741AF647 (Novus Biological) followed by flow cytometry.

### Stimulatory RNA production

DDO268, DDO268B, and the control X region expressing plasmid (with a T7 promoter) were linearized and *in vitro* transcribed with Hi Scribe T7 (NEB). RNA products were DNase treated and precipitated with LiCl. The OD260/OD280 ratios were between 2.00 and 2.25, and the OD260/OD230 ratios between 2.20 and 2.60. RNA purity and integrity were confirmed using an Agilent Bioanalyzer 2100. Endotoxin level was below 0.1 EU/mL/300 µg.

### Vaccine formulation

NP mRNA, NP mRNA/X region, NP mRNA/DDO268, and NP mRNA/DDO268B were encapsulated in the GenVoy Ionizable Lipid Mix (ILM) using a NanoAssemblr Ignite machine (Precision Nanosystems) following the manufacturer’s instructions. Encapsulation efficiency and RNA concentration were tested by Ribogreen Assay (Thermo Fisher). Final particle size was measured by DLS. mRNA and adjuvant RNA co-packaging was performed at a 1:1 molar ratio. All formulations contained the same total amount of RNA (about 0.5 µg total RNA per dose).

### Mice immunization

Mice were anesthetized with isoflurane and injected subcutaneously into a rear footpad. For toxicity studies, mice were injected with 50 µg of DDO268 or phosphate-buffered saline (PBS) at a final volume of 30 µL per dose. For immune response studies, mice were inoculated with BNT162b2 (0.125 µg); BNT162b2 (0.125 µg) + DDO268 (5 µg); BNT162b2 (0.125 µg) + RNA (5 µg); LNPs containing 1 ug IAV NP mRNA; LNPs containing 1 µg IAV NP mRNA/DDO268; LNPs containing 0.5 µg IAV NP mRNA; LNPs containing 0.5 µg IAV NP mRNA/X region; LNPs containing 0.5 µg IAV NP mRNA/DDO268; LNPs containing 0.5 µg IAV NP mRNA/DDO268B; empty LNPs or PBS at a final volume of 30 µL per dose. Leftover BNT162b2 SARS-CoV-2 vaccine was stored at −80°C prior to use in this study. Mice were primed and boosted 28 days later with the same vaccine formulations for adaptive immunity studies.

### Mice challenge

Mice were challenged intranasally with 40 TCID_50_ of IAV-PR8 39  days after boost. All mice were weighed daily post-challenge, and survival probability was recorded based on an increase or decrease in mortality.

### Complete blood count

Peripheral blood samples were collected from mice via cardiac puncture and blood was collected into EDTA-coated tubes to prevent clotting. Samples were processed immediately and analyzed using an automatic hematology analyzer HEMAVET. The results were generated automatically by the analyzer software and recorded.

### Blood chemistry analysis

Peripheral blood samples were collected from mice via cardiac puncture and collected into serum-separating tubes. Samples were processed immediately following standard protocols for each parameter using ACE Axcel Clinical Chemistry System.

### RT-qPCR

RNA was extracted from cells and tissue using TRIzol (Ambion Inc.) cDNA synthesis was performed with the High-Capacity cDNA Reverse Transcription kit (Applied Biosystems). For quantitative analysis by RT-PCR (qPCR), 10 ng/µL of cDNA was amplified using SYBR Green Mastermix (Thermo Fisher) in a Bio-Rad C1000 Touch thermal cycler (Bio-Rad). The primers used are listed in Table 1.

**TABLE 1:** Primer sequences used for quantitative PCR (qPCR)

Gene	Forward	Reverse	Species
GAPDH	5′-GCAAATTCCATGGCACCGT-3′	5′-CCACCACCCTGTTGCTGTAG-3′	Human
β-Actin	5′-AGAGCTACGAGCTGCCTGAC-3′	5′-CGTGGATGCCACAGGACT-3′	Human
*Ifnb1*	5′-GTCAGAGTGGAAATCCTAAG-3′	5′-ACAGCATCTGCTGGTTGAAG-3′	Human
*IL29*	5′-CGCCTTGGAAGAGTCACTCA-3′	5′-GAAGCCTCAGGTCCCAATTC-3′	Human
GAPDH	5′-CTCCCACTCTTCCACCTTCG-3′	5′-TCGCCCCACTTGATTTTGG-3′	Mouse
β-Actin	5′-AGGTGACAGCATTGCTTCTG-3′	5′-GCTGCCTCAACACCTCAAC-3′	Mouse
*Ifnb1*	5′-AGATGTCCTCAACTGCTCTC-3′	5′-AGATTCACTACCAGTCCCAG-3′,	Mouse
*Mx1*	5′-CAACTGGAATCCTCCTGGAA-3′	5′-GGCTCTCCTCAGAGGTATCA’3′	Mouse
*Il6*	5′-ACAGAAGGAGTGGCTAAGGA-3′	5′-CGCACTAGGTTTGCCGAGTA-3′	Mouse
*Il1b*	5′-TTGACGGACCCCAAAAGAT-3′	5′-GATGTGCTGCTGCGAGATT-3′	Mouse
*Il13*	5′-CCTCTGACCCTTAAGGAGCTTAT-3′	5′-CGTTGCACAGGGGAGTCT-3′	Mouse
*Cxcl10*	5′-CCTGCTGGGTCTGAGTGGGA-3′	5′-GATAGGCTCGCAGGGATGAT-3′	Mouse
*Ccl2*	5′-GCTTCTGGGCCTGCTGTTCA-3′	5′-AGCTCTCCAGCCTACTCATT-3′	Mouse
*TNFa*	5′-TCACTGGAGCCTCGAATGTC-3′	5′-GTGAGGAAGGCTGTGCATTG-3′	Mouse
*PR8 NP*	5′-CAGCCTAATCAGACCAAATG-3′	5′-TACCTGCTTCTCAGTTCAAG-3′	IAV virus

### Single-cell suspension

Footpads were processed following Fangzhou et al. (38) with modifications. Lymph nodes were digested using DNase (1 µg/mL) and Liberase (5 µg/mL) in RPMI 1640 Medium (Life Technologies) for 20 min at 37°C. Spleens were collected in RPMI 1640 Medium and dissociated. Lungs were inflated with 0.7 mL of digestion mix containing collagenase A (Sigma), dispase (Thermo Fisher), Liberase TL (Sigma), and DNAse I (Sigma) and incubated at 37°C for 30 min with agitation. Digested samples were filtered through a 70 µm mesh to obtain single-cell suspensions. Cells were washed with PBS containing 5% fetal bovine serum (FBS), treated with red blood cells lysis buffer (Sigma), and total viable cells were quantified with trypan blue staining using an automated cell counter (TC-20 Automated Cell Counter; Bio-Rad).

### Flow cytometry

Flow cytometry experiments were performed using a Cytek Aurora spectral flow cytometer (Cytek Biosciences), with at least 1 × 10^6^ events acquired. Data were analyzed using FlowJo V12 software (Tree Star Inc.). Single-cell suspensions were stained with fluorochrome-labeled antibodies. Fixable Viability Dye eFluor506 and monoclonal antibodies specific for mouse IFNγ (XMG1.2), Ly6c (HK1.4), CD3 (17A2), CD19 (eBio1D3), B220 (RA3-6B2), NK1.1 (PK136), CD11b (M1/70), and TNFα (MP6-XT22) were obtained from eBioscience. Monoclonal antibodies specific for mouse CD3 (17A2), CD4 (GK1.5), CD11a (H11578), XCR1 (ZET), PDCA1 (129 cl), CD11c (cN418), SIRPα (P84), CD64 (x54/7.1), CD44 (NIM-R8), CD62L (cMEL-14), CCR7 (4B12), CD103 (QA17A24) and MHC-II (M5/114.15.2) were obtained from BioLegend. Monoclonal antibodies for mouse CD86 (GL1), and CD8 (53-6.7) were obtained from BD BioSciences. For IAV-specific tetramers, H-2D^b^ tetramers bearing NP366-374 (ASNENMETM) and I-A^b^ tetramers bearing NP311-325 (QVYSLIRPNENPAHK) were obtained from NIH Tetramer Core Facility at Emory University. APC-labeled SARS-CoV-2 S-specific tetramer was MHC class I tetramer, residues 539–546, VNFNFNGL, H-2Kb. For intracellular staining, cells were fixed/permeabilized with the FoxP3/Transcription Factor Staining Buffer Set (eBioscience) and incubated with anti-IAV NP antibody conjugated with AlexaFluor-647 (Invitrogen).

### T-cell restimulation

For each sample, 1 × 10^6^ cells were restimulated using PMA (0.1 mg/mL) and ionomycin (1 mg/mL) or purified IAV NP protein (Sino Biological) for 4 h at 37°C followed by brefeldin treatment. Cells were resuspended in PBS with 1% FBS (Sigma) and blocked with anti-mouse FcψRIII/II (CD16/32; BD Biosciences) for 20 min on ice. T-cell subpopulations were defined using a surface marker panel, with activation determined by intracellular staining for IFNγ and TNFα.

### Quantification of influenza NP-specific serum antibodies

Sera from 21 post-boost immunized mice were analyzed for anti-SARS-CoV-2 Spike or anti-IAV NP IgG1 and IgG2c antibodies. ELISA plates (Nunc, MaxiSorp) were coated with 2 µg of purified SARS-CoV-2 Spike (kindly provided by Dr. Ellebedy, Washington University in St. Louis) or IAV-NP (Sino Biological) and treated with pre-diluted sera in triplicate, followed by HRP-conjugated anti-mouse IgG1 or IgG2c (Southern Biotech) and TMB substrate (Sera Care).

### Histopathology

Mice lungs were perfused and inflated with 0.7 mL of OCT compound (Tissue-Tek) mixed 1:1 (vol/vol) with 4% paraformaldehyde (Electron Microscopy Sciences) diluted in PBS. Inflated lungs were snap-frozen and stored at −80°C until sectioning. Tissue sections (4 µm) were stained with hematoxylin and eosin, and chronic lung disease was scored for peribroncholitis and goblet cell metaplasia. Area affected was determined, multiplied by the intensity scores previously defined, and the resulting weighted scores were graphed.

### IFN-γ, IL2 ELISpot assays

Double-color fluorescent ImmunoSpot (CTL) was used for the detection of primed IFN-γ-IL2-producing Th1-type memory T-cells. In brief, IAV NP or ionomycin/PMA (mitogen) were plated at the specified concentrations into capture antibody-precoated ELISpot assay plates. Then, 200,000 viable cells/well in 100 µL CTL-medium and cultured with the NP or mitogen for 24 h at 37°C and 9% CO_2_. After addition of detection antibody, the plates were scanned and spot-forming units were counted and analyzed using an S6 Ultra M2 Fluorospot reader. Spot numbers were automatically calculated using the Autogate function of the ImmunoSpot Software.

### Statistical analysis

Statistical significance was inferred using GraphPad Prism software version 10.0 (GraphPad Software, San Diego, CA). Unpaired *t*-test, one-way and two-way ANOVA with Bonferroni’s multiple comparison test were used. Log-rank Mantel-Cox test was used for survival. For animal experiments, group size consisted of 3–5 mice per group.

## Data Availability

Data are available upon request.

## SUPPLEMENTAL MATERIAL

Supplemental Figures mbio.03589-24-s0001.docx Figures S1 and S2.

Table S1 mbio.03589-24-s0002.docx CBC and chemistry profile of mice inoculated with DDO.

ASM does not own the copyrights to Supplemental Material that may be linked to, or accessed through, an article. The authors have granted ASM a non-exclusive, world-wide license to publish the Supplemental Material files. Please contact the corresponding author directly for reuse.
